# Feasibility of a Cardiac Scale in Measuring Blood Pressure

**DOI:** 10.1007/s12265-022-10243-y

**Published:** 2022-04-08

**Authors:** Daniel Yazdi, Sarin Patel, Kivanc Ozonat, Marat Fudim, Sarah Smith, Corey Centen

**Affiliations:** 1 Bodyport Inc, 970 Folsom Street, San Francisco, CA 94107 USA; 2 Department of Medicine, University of California San Francisco, 505 Parnassus Ave, San Francisco, CA 94143 USA; 3 Department of Medicine, Duke University Medical Center, Durham, NC USA; 4 Duke Clinical Research Institute, Durham, NC USA

**Keywords:** Blood pressure, Hemodynamics, Digital health, Remote monitoring

## Introduction

Blood pressure (BP) is a vital physiological measurement. Elevated BP affects over one-third of Americans and over 1 billion adults worldwide^1^. It is a risk factor for coronary artery disease, heart failure, chronic kidney disease, and stroke. Office BP measurements are limited by infrequency and confounders such as white coat hypertension. Home-based oscillometric BP improves accessibility but suffers from user and device error, as well as poor adherence. There is a need for simple, easy-to-use, home-based BP monitoring technologies. In this study, we investigated the accuracy of a connected digital scale with sensors that capture hemodynamic signals in measuring central mean arterial pressure (cMAP) compared to an FDA-approved reference device. cMAP is a measure of pressure in the ascending aorta and is a better predictor of adverse cardiovascular outcomes compared to brachial BP measurements^2^.

## Methods

**Fig. 1:**
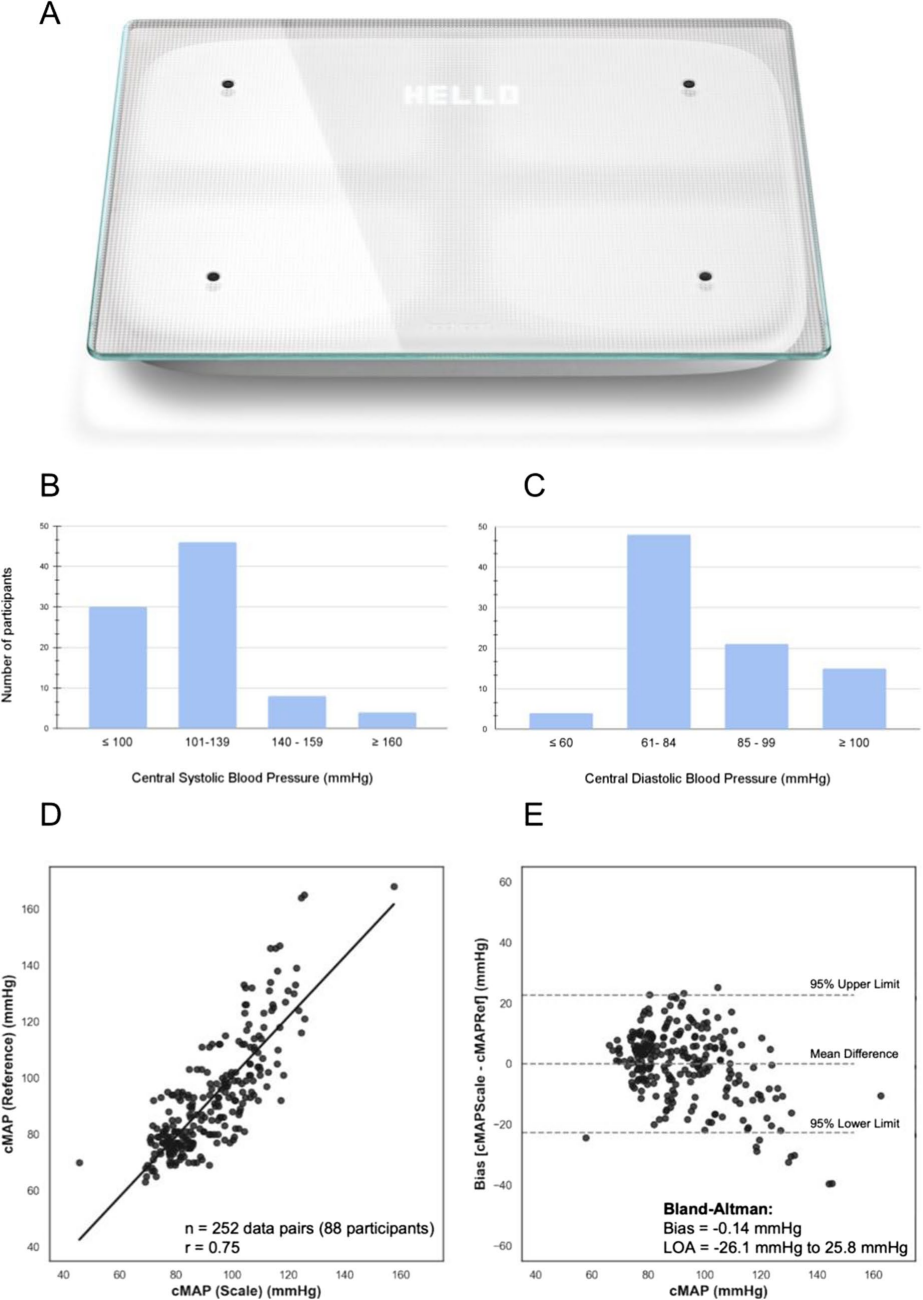
**A** Image of the Bodyport Cardiac Scale. **B**–**C** Histogram of the number of study participants versus the average central systolic and diastolic blood pressures (mmHg) per participant. **D** Scatter plot with a linear regression line for the scale-derived and reference device cMAP measurements (*n* = 252 data pairs from 88 participants, *r* = 0.75, MAPE = 9.6%). **E** Bland–Altman analysis plotting the bias (mean error of cardiac scale minus reference device) of − 0.14 mmHg and 95% limits of agreement of − 26.1, 25.8 mmHg (dotted lines)

This comparative, single-center, non-randomized, IRB approved study was conducted at the Clinimark Desaturation Laboratory at Avista Adventist Hospital in Louisville, CO, in March of 2017. Patients were recruited with the goal of meeting the BP distribution requirements in the ISO 81060–2 standard. A cMAP estimate was developed from biomarkers derived from the Bodyport Cardiac Scale (Bodyport Inc., San Francisco, CA) and the model’s performance was compared to the cMAP from a reference device, the FDA-cleared SphygmoCor XCEL Ⓡ (AtCor Medical, Naperville, IL). The cardiac scale has the physical appearance of a weight scale (Fig. 1A). It is embedded with ballistocardiography (BCG), impedance plethysmography (IPG), and electrocardiography (ECG) sensors that allow for the measurement of mechanical forces exerted by the heart, pulsatile blood flow, and electrical activity of the heart, respectively. The reference device is an FDA approved BP monitor that can measure central ascending aortic BP.

Guided by the ISO 81060–2 protocol, each participant contributed up to three simultaneous, paired measurements using the reference device and cardiac scale. Among the eighty-eight participants, 79, 6, and 3 participants contributed three, two, and one paired measurement(s), respectively, resulting in 252 paired measurements. Thirty-nine participants were female and 49 were male, with a mean age of 42 years and a standard deviation (SD) of 13 years. The mean (range) auscultatory brachial systolic and diastolic BPs in the study population were 124 mmHg (87–220) and 84 mmHg (58–139), respectively.

The BCG, IPG, and ECG signals from the cardiac scale allow for the measurement of several key physiologic parameters for estimating mean arterial pressure (MAP). The pre-ejection period (PEP) is the time between electrical depolarization and the start of ventricular ejection. A shorter PEP may reflect improved cardiac contractility. Pulse transit time (PTT), the time difference between the arrival of the electrical (ECG) and mechanical (BCG) signals, is related to arterial stiffness^3^. Furthermore, biomarkers such as the BCG amplitude are correlated with stroke volume and cardiac output^5^.

Using these biomarkers and other signal features as inputs, a lasso regression model was developed. The reference device measurements were compared to the output of the regression model. The performance of the model in calculating the Peasron correlation coefficient and Bland–Altman analysis was evaluated using the optimism-corrected performance bootstrapping technique with 1000 iterations^4^. The Bland–Altman analysis consists of measuring the mean error (bias) and the 95% limits of agreement (LOA) between the reference device and cardiac scale measurements. The 95% LOA is equal to the mean error ± 1.96 standard deviations (SD), and signifies a 95% probability the true mean error exists between those bounds.

## Results

The central systolic and diastolic BP distribution from the reference device is detailed in Fig. 1B–C. The mean cMAP from the reference device was 92 mmHg with a standard deviation of 20 mmHg. The cMAP derived from the cardiac scale and reference device had a Pearson correlation of 0.75 (95% confidence interval: 0.68–0.85, *p* < 0.001) and mean absolute percent error (MAPE) of 9.6% (Fig. 1D). Bland–Altman analysis resulted in a mean error (cardiac scale minus reference device) of − 0.14 mmHg, ± SD of 13.2 mmHg, and a 95% LOA of − 26.1 mmHg and 25.8 mmHg (Fig. 1E).

## Discussion

This study demonstrates the feasibility of using a cardiac scale with BCG, ECG, and IPG sensors to approximate cMAP. Limitations of this study include a small sample size, along with intrinsic errors of both the reference BP measurement and scale technology. Future studies can include a higher incidence of hypertension, improved feature selection for model development, along with analysis of longitudinal BP measurements with clinical perturbations. External validation studies would also be beneficial.

There may be significant clinical utility in using a cardiac scale for longitudinal BP monitoring. The cardiac scale would likely enhance the ease and frequency of use compared to traditional oscillometric techniques. In addition to measuring BP, this same step can be used to obtain other hemodynamic biomarkers (e.g., stroke volume, cardiac output, heart rate) from the scale, potentially reshaping how we longitudinally and remotely care for patients^5^.

## Supplementary Information

Below is the link to the electronic supplementary material.

Supplementary file1 (DOCX 285 KB)

## Abbreviations

BCG: Ballistocardiography
BP: Blood pressure
cMAP: Central mean arterial pressure
ECG: Electrocardiography
IPG: Impedance plethysmography
LOA: Limits of agreement
MAP: Mean arterial pressure
MAPE: Mean absolute percentage error
SD: Standard deviation
PEP: Pre-ejection period
PTT: Pulse transit time

## References

[1] Writing Group Members *et al.* Executive summary: Heart disease and stroke statistics--2016 update: A report from the American heart association. *Circulation* **133**, 447–454 (2016). 10.1161/CIR.0000000000000366 26811276

[2] Roman MJ (2009). High central pulse pressure is independently associated with adverse cardiovascular outcome the strong heart study. Journal of the American College of Cardiology.

[3] Geddes LA, Voelz MH, Babbs CF, Bourland JD, Tacker WA (1981). Pulse transit time as an indicator of arterial blood pressure. Psychophysiology.

[4] Steyerberg, E. W. Overfitting and optimism in prediction models. in *Clinical prediction models: A practical approach to development, validation, and updating* (ed. Steyerberg, E. W.) 95–112 (Springer International Publishing, 2019).

[5] Yazdi, D. *et al.* Noninvasive scale measurement of stroke volume and cardiac output compared with the direct Fick method: A feasibility study. *J. Am. Heart Assoc.* **10**, e021893 (2021). 10.1161/JAHA.121.021893 PMC9075258 34873927

