# Supplementary material for: Feasibility of a Cardiac Scale in Measuring Blood Pressure
Source: J Cardiovasc Transl Res. 2022 Apr 8;15(5):1212–4. doi: 10.1007/s12265-022-10243-y (PMC9622550; doi:10.1007/s12265-022-10243-y)
Supplement: Supplementary file 1 — Supplementary file1 (DOCX 285 KB) [file 12265_2022_10243_MOESM1_ESM.docx]

Funding: This study was funded by Bodyport Inc.

Conflict of Interest: Daniel Yazdi, Sarin Patel, and Kivanc Ozonat are employees at Bodyport Inc. Marat Fudim consults for Bodyport Inc. Sarah Smith and Corey Centen are co-founders of Bodyport Inc.
